# Modeling the Spatiotemporal Habitat Suitability Distributions of Cultural Keystone *Bidens macroptera* (Adey Abeba) Under Climate Change Scenarios in Ethiopia

**DOI:** 10.1002/ece3.72115

**Published:** 2025-09-06

**Authors:** Tsige Hailegiorgis, Debissa Lemessa, Daniel Melese, Mikiyas Abebe, Sileshi Nemomissa

**Affiliations:** ^1^ Department of Plant Biology and Biodiversity Management Addis Ababa University Addis Ababa Ethiopia; ^2^ Department of Biology Kotebe University of Education Addis Ababa Ethiopia; ^3^ Department of Biology Mizan Tepi University Tepi Ethiopia; ^4^ Department of Biology Woldia University Woldia Ethiopia

**Keywords:** *Bidens macropetra*, climate change, cultural species, ensemble modeling, Ethiopia, habitat suitably range

## Abstract

Bidens macroptera symbolizes the change of a season, marking the transition from the rainy season to autumn, heralding the new year for Ethiopians. Despite a general understanding of its geographic regions, significant gaps remain in identifying the habitat distribution and key predictor variables of Bidens macroptera through species distribution modeling (SDM) in the context of climate change. We developed an ensemble species distribution model using 2 statistical and 3 machine learning algorithms. We collected 119 presence and pseudoabsence points to train and validate bioclimatic variables through 5‐fold cross‐validation with the “SDM” package in R software. We calculated the Variance Inflation Factor (VIF) to assess multicollinearity among environmental variables. Projections were made for medium (SSP 2–4.5) and extreme (SSP 5–8.5) greenhouse gas emissions for the periods of the 2050s and 2070s. The performance of the models was evaluated by Area Under the Curve (AUC) and True Skill Statistic (TSS). A weighted average threshold value at the sum of sensitivity and specificity of the TSS was used to classify habitat suitability using ArcGIS 10.4. The ensemble model showed strong performance, with an AUC ranging from 0.96 to 0.98 and a TSS of 0.84–0.92. Individually, MaxEnt outperformed with an AUC of 98% and a TSS of 92%. The mean temperature of the driest quarter (Bio 9) emerged as the most influential, followed by soil pH and slope. With the current climate conditions, 89.81% of habitats are classified as unsuitable, while 5.64% are least suitable, 2.46% moderately suitable, and 2.09% highly suitable. However, all future projections revealed a decline in suitable habitats, increasing the risk of local extinction. Therefore, it is essential to develop a conservation plan and strengthen climate change adaptation strategies to mitigate habitat loss for this iconic highland species.

## Graphical abstract

*Bidens macroptera* marks the transition from the rainy season to autumn, signifying the new year for Ethiopians. Its distribution is crucial for conservation, but data on its current status and future dynamics are limited due to climate change. Our modeling shows a high percentage of unsuitable habitats and projected declines, underscoring the urgent need for a conservation plan to address habitat loss.

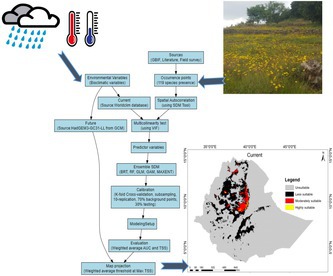

## 1 Introduction

Ethiopia is a biodiversity hotspot in East Africa, rich in endemic and threatened plant species (Fashing et al. 2022; Kidane et al. 2019). Among its approximately 6000 vascular plants, around 627 species are endemic (Demissew et al. 2021). These days, climate change is altering the natural distribution of endemic, nearly endemic, and native plants due to rising temperatures and fluctuations in precipitation patterns. These conditions are leading to an increase in the relative abundance of drought‐tolerant species while reducing the abundance of water‐demanding species. Furthermore, the increase in temperature is predicted to cause a northward expansion of the suitable growing habitat (Craufurd and Wheeler 2009) and disturb the life cycle of many species (Feeley et al. 2020). The annual average maximum temperature in Ethiopia has increased by approximately 1°C since 1950 (Funk et al. 2012). Furthermore, precipitation patterns have become more erratic, a trend linked to climate change (Zegeye 2018).

Globally, numerous researchers have utilized Species Distribution Modeling (SDM) to examine how climate change influences species distributions (Sinclair et al. 2010; Zimmermann et al. 2010). In Ethiopia, some SDM studies indicate that endemic and native plant species, particularly those adapted to highland environments, are experiencing a reduction in their distribution (Abebe et al. 2024; Yebeyen et al. 2022). These species have limited options for shifting their distribution to higher elevations. Conversely, other studies suggest that some invasive and temperature‐tolerant species may expand their ranges (Ahmed et al. 2021; Teklegiorgis et al. 2024). Therefore, utilizing SDM information is essential for guiding conservation efforts and managing biodiversity, especially for culturally significant plants like *Bidens macroptera* in Ethiopia.

*Bidens macroptera* (Sch.Bip. ex Chiov.) Mesfin is a plant species endemic to the Horn of Africa, specifically found in Ethiopia and Eritrea (Tadesse 1993). “Abebayehush” (have you seen the flowers) is a typical traditional song by young girls during the Ethiopian New Year (Enkutatash) to mark the upcoming mass flowering of this species. This plant signals the end of the rainy season and the beginning of Tseday (autumn) in Ethiopia. The mass flowering of this plant creates a unique mountainous landscape covered in variegated yellow, clearly seen from distant viewpoints (Siebert and Ramdhani 2004). Historically, Ethiopian farmers also use this mass flowering event to predict the duration of the rainy season and the end of the main farming season, known as “Meher.” Moreover, it is highly valued and commonly used for celebratory events, such as the bouquets prepared for Meskel Day, which Orthodox Christians believe commemorates the discovery of the “True Cross” in late September (Antohin 2019; Kaplan 2021). In Ethiopian Orthodox Christianity, there is a belief that after Queen Sheba (Makeda) visited King Solomon, she returned to Ethiopia with great wisdom and gifts (Spencer 2024). This event is often linked to the time of blooming of *B. macroptera*, and it was presented to her (Goshu and Woldemariam 2024). These show the deep connections of this plant to the culture, nature, and religious figures of Ethiopia. Furthermore, *B. macroptera* provides vital ecosystem services, such as food sources for insects, particularly honeybees and birds, and materials for religious painting and contemporary dyeing in Ethiopia (Gebreyohans and Gebremariam 2017; Gizaw et al. 2020; Shegaw and Giorgis 2021; Tulu et al. 2023).

*Bidens macroptera* predominantly thrives in the Ethiopian highlands, where it grows in a variety of habitats, including rocky mountain slopes, upland grasslands, ericaceous shrublands, and forest edges. Although it occasionally appears along roadsides, it is more commonly associated with natural and semi‐natural high‐altitude ecosystems. Despite a general understanding of its geographic regions, significant gaps remain in identifying the habitat distribution of *B. macroptera* through species distribution modeling (SDM). Additionally, key predictor variables from bioclimatic, topographic, lithologic, and anthropogenic factors need to be determined to better understand species distribution (Marshall et al. 2021; Schmitt et al. 2013). The main objective of this study is to investigate the habitat distribution and key predictor variables of *B. macroptera* using species distribution modeling (SDM) in the context of climate change. To achieve this, we addressed the following questions: (1) What is the ideal habitat range for *B. macroptera* in the context of climate change? and (2) Which environmental variables determine its distribution? The results of this study will provide insights into where *B. macroptera* can thrive and be abundantly distributed, thereby aiding in effective conservation planning efforts.

## 2 Methods

### 2.1 Study Area

The study was conducted in Ethiopia, which lies between latitudes 3° and 15° N and longitudes 33° and 48° E (Figure 2). The area of the country is about 1.13 million km^2^, and its altitude ranges from 125 m below sea level at the Danakil/Afar Depression to 4620 m a.s.l. at Mt. Ras Dashen. Ethiopia has mosaic topography, and the Great Rift Valley divides Ethiopia into northwestern and southeastern land masses that comprise different vegetation types (Friis et al. 2010). The country has average annual temperatures between −5°C and over 32°C, with precipitation levels varying from 250 mm to more than 2,700 mm (Berhane 2018; Fazzini et al. 2015).

**FIGURE 1:**
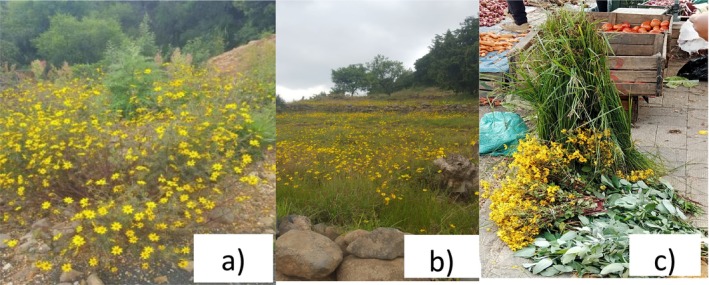
*Bidens macroptera*: (a) habit; (b) landscape; and (c) at the street market in Addis Ababa, Ethiopia.

**FIGURE 2:**
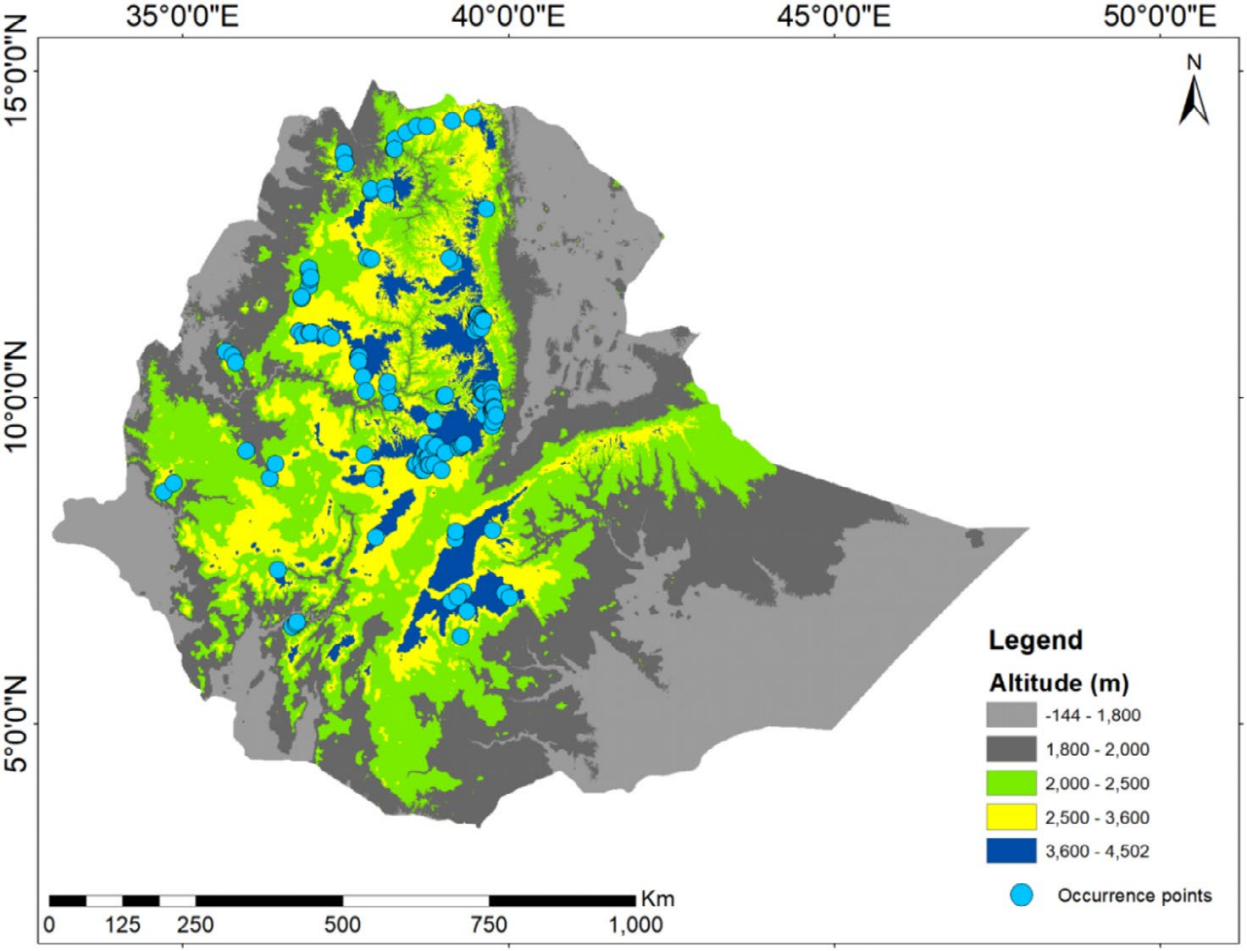
Occurrence points of *Bidens macroptera* by altitude (m).

*Bidens macroptera*, commonly known as Adey Abeba (in Amharic), is a perennial herb in the Asteraceae family that typically reaches a height of 60–100 cm. The stems arise from a woody root stock and exhibit decumbent to erect growth habits. The leaves have a dark olive‐green upper surface and a pale, slightly fleshy underside. The flower is a key identification feature, widely recognized for its yellow hue that brightens many landscapes across the country (Figure 1). The flower heads are cymose inflorescences. The ray florets are yellow with orange blotches placed centrally in the middle and at the base. The disc floret corolla is tubular. *Bidens macroptera* typically grows at an altitudinal range of 1750 m (rarely), 2000–3700 m, and abundantly at 2500–3700 m (Chiemela et al. 2018; Fichtl and Adi 1994; Tadesse 1993, 2004) (Figure 2).

### 2.2 Species Occurrence Data

Georeferenced occurrence points for *B. macroptera* were collected from the Global Biodiversity Information Facility (www.gbif.org) database (*n* = 6, listed as preservation), published literature (*n* = 7, with specimen collections in ETH (Ethiopian National Herbarium)), and field surveys (*n* = 135). In the field survey, sample points were systematically collected along major roads to districts in Ethiopia, extending 5–10 km to either side at 5 km intervals. A random point was recorded within a random 20 m^2^ subquadrant in each 5 km^2^ area. The survey took place from July to November in 2023 and 2024, utilizing mobile GIS technology.

We employed spatial rarefaction to assess the autocorrelation of occurrence data points in space, revealing that nearby points often exhibit similar characteristics, such as physical traits or species occurrences. Many SDMs rely on direct sources of species occurrence points from databases like GBIF, along with randomly collected data. However, these datasets are often not systematically collected and can vary in resolution, potentially introducing modeling bias by violating the assumption of independent and identically distributed sampling points. To address this issue, we utilized the Spatially Rarefy Occurrence Data Tool from the SDM Toolbox (v. 2.5) (Brown 2014) in combination with ArcGIS (v. 10.4). This tool effectively filters closely spaced occurrence points within a defined grid, retaining only one occurrence record within a given area. In this study, we rarefied all three sources of occurrence points into 5 km^2^ raster cells. By reducing redundancy and creating a more uniform sampling plot, this process enhances the representativeness of the dataset and minimizes bias in modeling (Boria et al. 2014), thereby improving the reliability of model outputs. Finally, we used 119 occurrence points for modeling (Figure 2).

### 2.3 Climate Data

Bioclimatic variables are employed in species distribution modeling (SDM) to identify climatic niches based on occurrence records and project these into geographic space. The bioclimatic data were derived from the WorldClim dataset (Hijmans et al. 2005, http://www.worldclim.org), which is used to determine the distribution of each species (Pearson and Dawson 2003). These data were interpolated from climate information collected between 1950 and 2000. We also utilized two soil variables (soil type and soil pH [0–20 cm scaled]) from ISRIC—World Soil Information (https://www.isric.org) at a resolution of 250 m^2^, which are critical for species distribution (Dubuis et al. 2013; Ni and Vellend 2024). Additionally, we incorporated topographic variables (elevation, slope, and aspect) at a resolution of 90 m^2^, sourced from the Shuttle Radar Topography Mission (SRTM) digital elevation model (DEM) accessed through USGS Digital Elevation (http://earthexplorer.usgs.gov). Land cover data was obtained from the Copernicus Climate Data Store (https://cds.climate.copernicus.eu/) at a resolution of 100 m^2^. These environmental data, obtained from various scales, were resampled by aggregation to align with the bioclimatic spatial resolution of 30 arc‐seconds (approximately 1 km^2^). These environmental variables often exhibit a higher degree of collinearity, which can lead to misleading model performance (De Marco and Nóbrega 2018). To address this challenge, we employed the Variance Inflation Factor (VIF), which quantifies collinearity by calculating the square of the multiple correlation coefficient for each predictor in the model (O'Brien 2007). We used a threshold for VIF of less than 5. We utilized the ‘usdm’ package in R (version 4.4.1) for these calculations (Naimi and Araújo 2016; Pradhan and Setyawan 2021). This analysis reduced our set of environmental predictors from 25 to 11 by excluding variables with VIF values exceeding this threshold (Table 1).

**TABLE 1:** Current predictor variables after excluding the collinear factors.

Environmental variables	Predictor variables	Code	Unit	VIF < 5
Bioclimatic	Isothermality (Bio_2/Bio_7) (ratio of mean diurnal range to temperature annual range)	Bio_3	—	2.131448
	Temperature annual range	Bio_7	°C	1.788433
	Mean temperature of the driest quarter	Bio_9	°C	2.867111
	Precipitation of the driest month	Bio_14	mm	2.747686
	Precipitation seasonality (coefficient of variation)	Bio_15	—	2.477502
	Precipitation of the warmest quarter	Bio_18	mm	2.084749
	Precipitation of the coldest quarter	Bio_19	mm	2.818612
Topographic	Slope		°	1.212367
Lithologic	Soil pH		pH	4.091481
	Soil type		—	1.494922
Antropogenic	Land cover		—	2.668010

Spatiotemporal projections of bioclimatic variables were used to predict how species distributions may evolve during the periods of 2041–2060 (2050s) and 2061–2080 (2070s) based on current species distribution models. This forecasting depends on scenarios from the latest iteration of the Coupled Model Intercomparison Project Phase 6 (CMIP6) (Eyring et al. 2016), sourced from the Intergovernmental Panel on Climate Change's (IPCC) sixth assessment report (Masson‐Delmotte et al. 2021). The models were downloaded under two scenarios: moderate development (SSP 2–4.5) and high emission (SSP 5–8.5), both characterized by increased greenhouse gas concentrations. The future projections data were sourced from the Global Circulation Model (GCM) particularly from HadGEM3‐GC31‐LL, developed by the UK Hadley Centre for Climate Prediction and Research. This model operates under the latest coupled configuration (Williams et al. 2018) and ranks among the top three GCMs, alongside EC‐Earth3 Veg and EC‐Earth (Nguyen‐Duy et al. 2023).

### 2.4 Model Selection, Calibration, and Validation

We employed an ensemble modeling approach, a statistical technique that enhances forecast accuracy by combining predictions from multiple models (Doblas‐Reyes et al. 2005). In addition, it minimizes uncertainty and bias by utilizing a diverse range of models instead of relying on a single best model (Di Napoli et al. 2020; Parker 2013). In our analysis, we utilized five individual models from different algorithms including Random Forest (RF), Generalized Linear Model (GLM), Generalized Additive Model (GAM), Boosted Regression Trees (BRT), and Maximum Entropy (MaxEnt) (Figure 2). RF and BRT are effective for capturing complex relationships, with RF using an ensemble of decision trees to reduce overfitting and enhance predictive reliability (Elith et al. 2008; Iverson et al. 2008), while BRT sequentially corrects errors from previous trees (Sharma and Sahoo 2022; Wang et al. 2024). Both algorithms excel in handling nonlinear relationships and provide valuable insights into variable importance (Hussain et al. 2021). GLM offers flexibility for various response variables, making it suitable for diverse ecological datasets (Guisan et al. 2002), and GAM is effective in modeling nonlinear effects (Dormann et al. 2013). Additionally, GLM and GAM strike a balance between interoperability and flexibility (Xie 2024). MaxEnt is particularly suited for presence‐only data, applying the principle of maximum entropy to forecast species distributions based on environmental factors (Merow et al. 2013). The ensemble modeling was achieved using the “dismo” package in the R environment, effectively reducing biases inherent in individual models (Merow et al. 2016; Pecchi et al. 2019).

We calibrated the models based on training data using 119 presence records and 10,000 background points (Elith et al. 2011). The modeling process involved *K*‐fold Cross‐validation (*K* = 5) using “dismo” package, and presence data was split into 70% for training and 30% for testing. To minimize the impact of random variations, the model was run ten times, resulting in 50 distinct calibrated models. This approach also helps reduce potential bias introduced by resampling.

**FIGURE 3:**
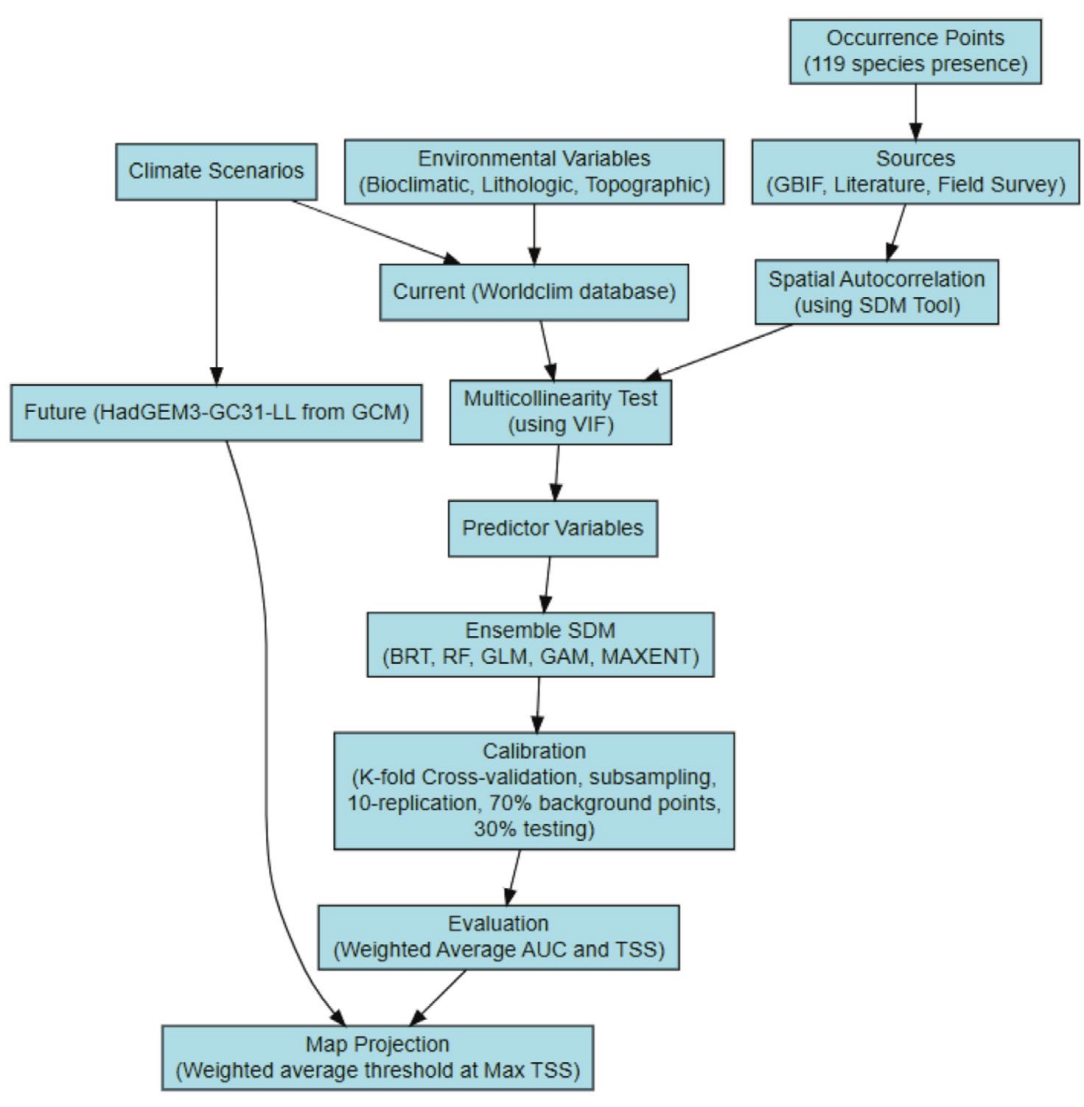
Overview of the data integration workflow for ensemble SDM.

Model validation was employed by a weighted ensemble modeling approach that assigns weights based on AUC (Area Under the Curve) and TSS (True Skill Statistic) scores (Shabani et al. 2016). AUC is a threshold‐independent metric that indicates the model's ability to distinguish between where a species is predicted to be present and absent (Swets 1988). The AUC ranges from 0 to 1 and measures model performance. An AUC > 0.9 is excellent, 0.8–0.9 is good, 0.7–0.8 is fair, 0.6–0.7 is poor, and 0.5–0.6 is bad, and AUC < 0.5 is not better than random prediction (Fourcade et al. 2018). TSS is a threshold‐dependent metric that considers both sensitivity (true positive rate) and specificity (true negative rate), with values ranging from −1 to 1. A TSS of 1 indicates perfect prediction, 0 denotes random chance, and negative values suggest performance worse than random guessing. TSS > 0.75 is excellent, while 0.40 < TSS < 0.75 is good, and TSS < 0.40 is poor (Eskildsen et al. 2013). To integrate the R results with ArcGIS, the writeRaster function from the raster package was used. This function exports raster data, including predictions, in GeoTIFF format, enabling further analysis and visualization within the ArcGIS environment. The importance of different climatic factors was assessed and ranked according to their weighted average contributions (TSS and AUC) across all models, helping to identify which variables are most influential. Generally, graphical illustrations of the overall methodological procedures used for this study are presented in Figure 3.

### 2.5 Habitat Suitability Classification

The ensemble model produced a prediction map of species richness using binary data to indicate species presence or absence. To convert this map into binary outcomes, we optimized a weighted average threshold setup that maximizes sensitivity and specificity, improving the model's ability to identify suitable habitats (Lu et al. 2012). We categorized the predicted values into four habitat suitability levels: unsuitable (0–0.2), least suitable (0.2–0.4), moderately suitable (0.4–0.6), and highly suitable (above 0.6) (Hao et al. 2020). In addition, to evaluate changes across different scenarios for both suitable and unsuitable habitat classes, we computed the gains, losses, and remaining habitats (Appendix A).

## 3 Results

### 3.1 Performance of Modeling Algorithms

All SDM algorithms demonstrated excellent performance in terms of both AUC and TSS. The ensemble model showed strong performance, with an AUC ranging from 0.96 to 0.98 and a TSS of 0.84–0.92 (Table 2). Among the individual models, MaxEnt achieved the highest scores with an AUC of 0.98 and a TSS of 0.92, while the BRF model demonstrated the lowest performance.

**TABLE 2:** Performance evaluation of each SDM using different statistical parameters.

Scenario	Statistics	BRT	RF	GLM	GAM	MaxEnt
Current (base scenario)	AUC	0.96	0.98	0.96	0.98	0.98
	Cor	0.57	0.63	0.51	0.56	0.57
	TSS	0.84	0.90	0.88	0.91	0.92
	Threshold (Wa)	0.031	0.054	0.035	0.028	0.099
	Deviance	0.04	0.04	0.04	0.04	0.11

The ensemble model achieved an AUC ranging from 0.941 to 0.958 derived from ROC curves, reflecting an excellent balance between sensitivity and specificity. It effectively identifies suitable and unsuitable areas across various thresholds (weighted average, Wa). Notably, the Random Forest (RF) model's curve is closest to the top‐left corner, demonstrating superior balance compared to other algorithms (Appendix B).

### 3.2 Relative Contribution of Predictor Variables

**FIGURE 4:**
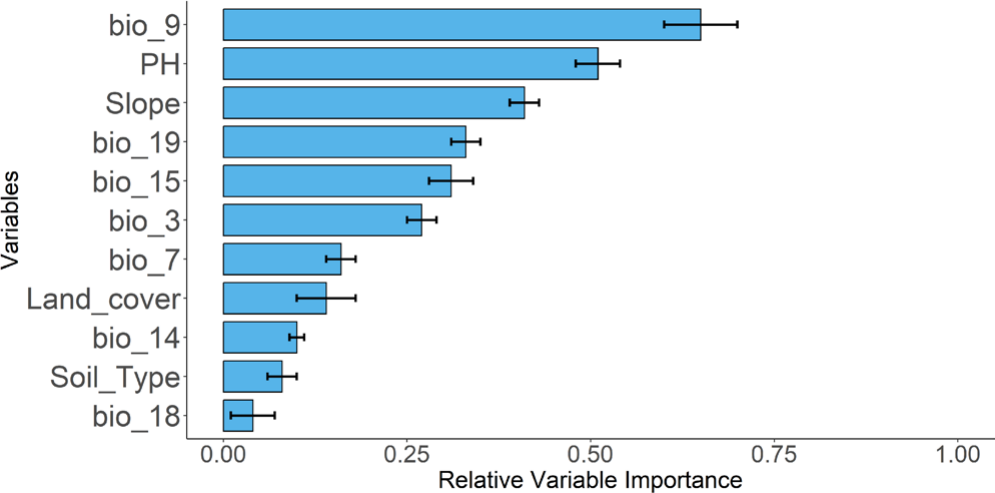
Contribution of relative variable importance of variables for current scenario.

Among the predictor variables, the mean temperature of the driest quarter (Bio 9) emerged as the most influential, followed by soil pH and topographic variables, particularly slope. Precipitation of the warmest quarter (Bio 18) and soil type were the least influential in determining the habitat distribution of *B. macroptera* (Figure 4).

### 3.3 Potential Habitat Suitability

Currently, *B. macroptera* is distributed across an area of 115,434.40 km^2^ (Table 3) out of a total of 1,133,191.57 km^2^. Within the study area, 89.81% is classified as unsuitable for *B. macroptera* distribution, while only 10.19% is considered suitable. Within the total suitable area for *B. macroptera*, the model indicated that 5.64%, 2.46%, and 2.09% were categorized as least, moderately, and highly suitable, respectively. The most suitable habitat is located in Ethiopia's central highlands, specifically the dry afromontane vegetation types.

**TABLE 3:** Habitat suitability coverage across different scenarios.

Scenarios		Habitat Suitability							
		Unsuitable		Least suitable		Moderately suitable		Highly suitable	
		km^2^	%	km^2^	%	km^2^	%	km^2^	%
Current	—	1017763.61	89.81	63866.81	5.64	27850.36	2.46	23717.23	2.09
SSP_4.5	50s	1050651.16	92.72	39008.94	3.44	32262.08	2.85	11276.05	1.00
	70s	1054177.48	93.03	30855.91	2.72	30912.21	2.73	17252.59	1.52
SSP_8.5	50s	1069929.94	94.42	28412.50	2.51	21398.25	1.89	13457.50	1.19
	70s	1077548.52	95.09	22973.04	2.03	18209.66	1.61	14468.45	1.28

**FIGURE 5:**
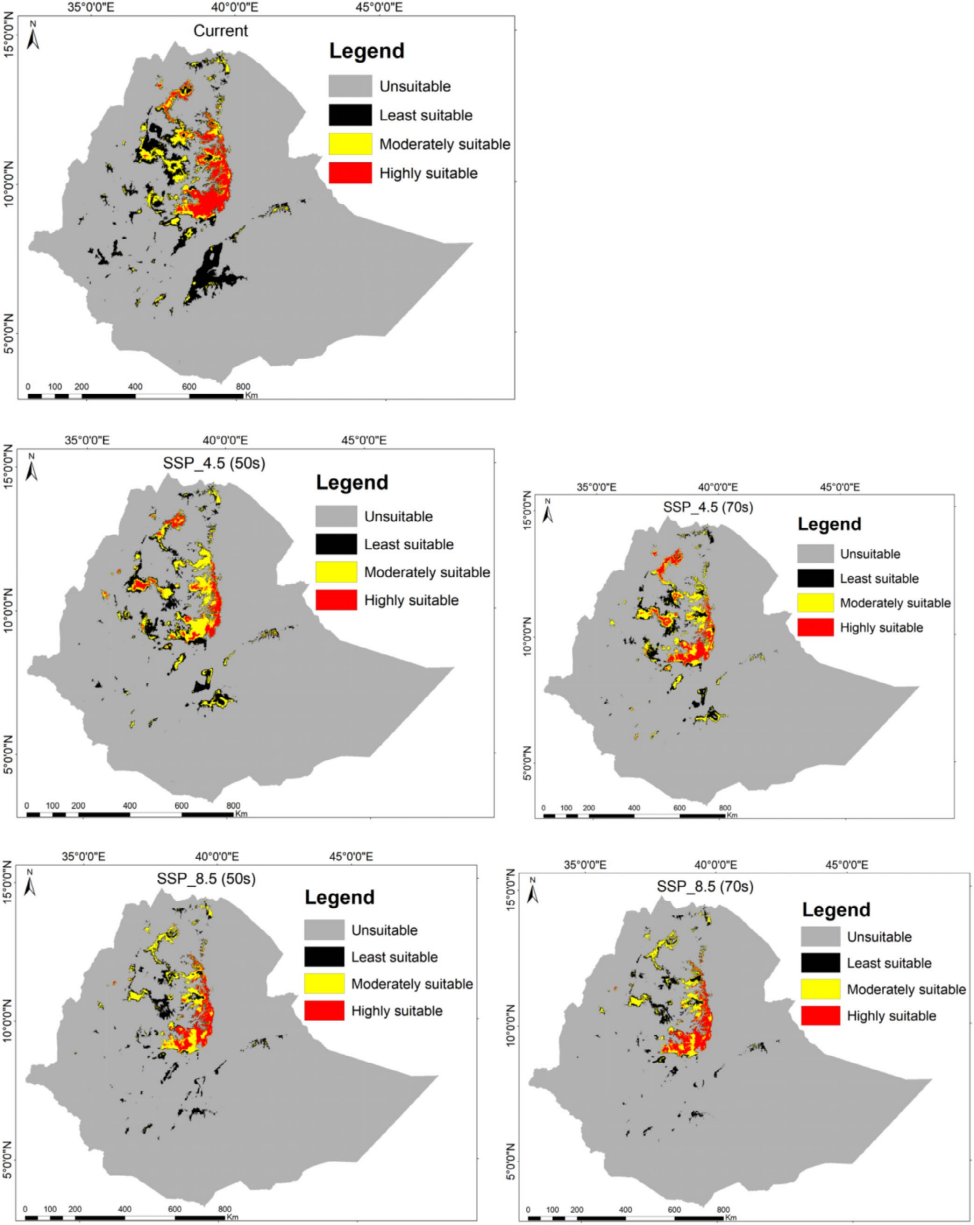
Distribution and habitat suitability categories of *Bidens macroptera* under various climate scenarios.

Under future climate scenarios, the distribution of *B. macroptera* is projected to decline in all suitability classes. Our analysis shows a decreasing trend in the area of suitable habitat among the various categories of suitable habitat, with least suitable areas diminishing more rapidly than highly suitable areas over time (up to ~50%) (Figure 5).

### 3.4 Habitat Suitability Change

**FIGURE 6:**
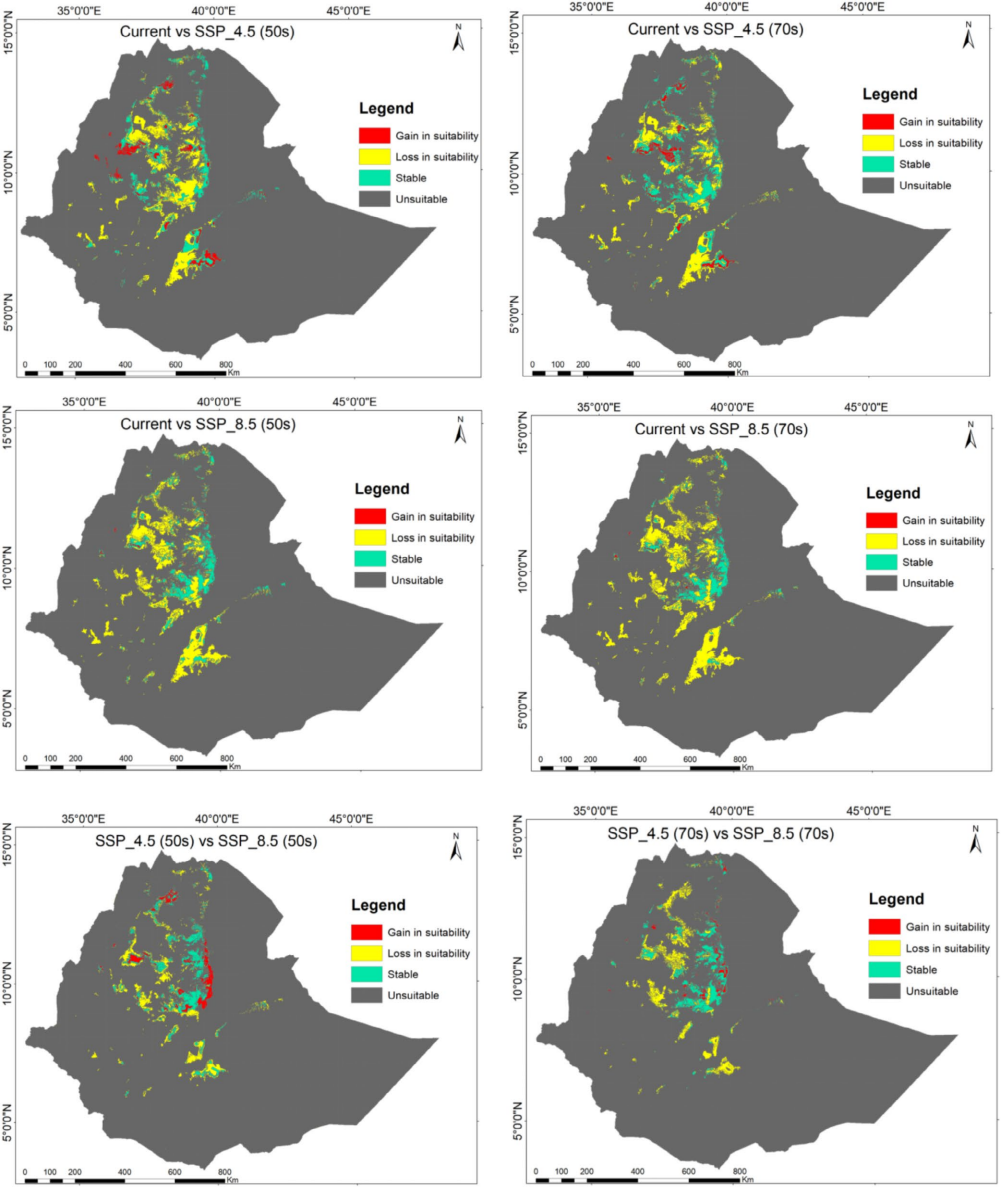
Gain, loss, and remain of suitable and unsuitable habitats across different scenarios.

In the SSP 4.5 and SSP 8.5 scenarios, suitable habitats for *B. macroptera* decreased compared to current climatic conditions, with a loss of up to 7.46%. The reduction in suitable habitat was particularly significant under the worst carbon emission scenarios. Conversely, unsuitable habitat increased across all scenarios (Table 4 and Figure 6).

**TABLE 4:** Change of habitat suitability among different carbon emission scenarios.

Suitability assessment category	Current/SSP_4.5		Current/SSP_8.5		SSP_4.5/SSP_8.5	
	2050s	2070s	2050s	2070s	2050s	2070s
Gain in suitability	14661.94	12637.80	1873.97	1963.07	15523.44	7519.46
%	1.29	1.12	0.17	0.17	1.37	0.66
Loss in suitability	65441.98	58352.80	78182.91	84549.19	36416.20	40994.89
%	5.77	5.15	6.90	7.46	3.21	3.62
Stable	39272.72	45786.75	35621.82	29038.07	34646.62	32106.25
%	3.47	4.04	3.14	2.56	3.06	2.83
Unsuitable	1013821.37	1016420.11	1017518.75	1017646.44	1046611.42	1052576.92
%	89.47	89.69	89.79	89.80	92.36	92.89

## 4 Discussion

This study provides a comprehensive evaluation of the current and future distribution of *B. macroptera* based on key environmental variables. We identified a total of 119 occurrence points for this culturally iconic and ecologically important highland species in Ethiopia. Of these points, 97% were newly collected, adding critical distribution data for this species.

### 4.1 Modeling Performance of Algorithms

The ensemble modeling approach demonstrated robust predictive performance (AUC 0.96–0.98; TSS 0.84–0.92), reinforcing the reliability of the findings. These results align with the excellent modeling performance reported by Fourcade et al. (2018) and Swets (1988). Even though the values from each model were consistent, slight similarities were observed; the performance of MaxEnt was somewhat higher in the ensemble modeling approach. This is because MaxEnt typically achieves higher predictive accuracy than other models, as demonstrated by various studies (Hao et al. 2020; Kaky et al. 2020; Valavi et al. 2022). In addition, the Random Forest (RF) model demonstrated the highest performance in the ROC curve analysis. This indicates its effectiveness in classifying geographic areas as either suitable or unsuitable (Zhang et al. 2019).

### 4.2 Environmental Factors Affecting *B. macroptera* Distribution

The most important predictor variables in our SDMs were bioclimatic factors (temperature), lithophytic factors (soil pH), and topographic factors (slope) (Figure 5). However, precipitation variables showed relatively low importance, likely due to collinearity with elevation, slope, and temperature, which led to their removal during the multicollinearity test. Additionally, land cover was less significant, as this plant primarily thrives in natural habitats, particularly in mountainous regions less accessible to human disturbance.

The mean temperature of the driest quarter (Bio 9) was the most influential variable, indicating the species' sensitivity to thermal conditions during its critical growing period, as noted by Bykova et al. (2012). Habitat temperature also affects other variables like precipitation and soil properties, which are fundamental for the species' stable ecology (Lembrechts et al. 2019). As a highland climate plant, *B. macroptera* is adapted to cooler temperatures and has a lower growth threshold. During our collection, we observed that it does not grow in saturated soils; rather, it requires a continuous water supply while preferring well‐drained soils on mountain slopes. Additionally, soil pH was identified as a crucial factor in the distribution of *B. macroptera*. This suggests that the plant requires optimal soil pH to improve habitat suitability and ensure ideal growth conditions, consistent with previous studies (Buri et al. 2017; Dubuis et al. 2013). Topography, particularly slope, is also critical, aligning with the species' ecological preference for well‐drained and high‐elevation terrains. As you move up a steep slope, the elevation increases (Krömer et al. 2013). Therefore, considering the impact of these environmental variables on the distribution of *B. macroptera* is crucial for the conservation and restoration of species in their natural habitats (Ab Lah et al. 2021; Hirzel and Le Lay 2008), as well as for in situ conservation.

### 4.3 Current Distribution of *B. macroptera*

The results reveal that only 11% of Ethiopia currently offers suitable habitat for the species, with highly suitable zones constituting a mere 2%. *Bidens macroptera* is mainly found in dry afromontane forests and grasslands, extending into afroalpine regions. This distribution occurs in the highland provinces of Showa, Tigray, Gondar, Gojam, Wollo, Kefa, Gamogofa, and Bale Harerge, characterized by mountainous terrain, rocky slopes, grasslands, forest edges, and open ericaceous scrub (Fichtl and Adi 1994; Tadesse 2004). The observed distribution aligns with current modeling results, confirming predictions about suitable habitats for the species.

### 4.4 The Projected Future Distribution of *B. macroptera*

The current distribution of *B. macroptera*, which already occupies a limited ecological niche, is expected to shrink further under moderate (SSP4.5) and extreme (SSP8.5) climate scenarios. Unlike some species that may benefit from climate change (Liu et al. 2017), *B. macroptera* is likely to face negative consequences (Pacifici et al. 2015). The species may also be forced to shift to higher elevations (Kelly and Goulden 2008; Lenoir et al. 2008; Wilson et al. 2005), potentially displacing it from its original habitat and likely leading to extinction due to elevational constraints (Carlson et al. 2017).

Plants with specific habitat requirements are generally more vulnerable to climate change than those with broader ranges (Manes et al. 2021). Importantly, future projections indicate that *B. macroptera* lacks the adaptive flexibility to shift downslope or into human‐modified landscapes, making it especially susceptible to climate‐driven habitat loss. Furthermore, recent harvesting of *B. macroptera* during its flowering stage for ornamental purposes has resulted in fewer viable seeds for future growth. This anthropogenic effect, primarily occurring in Ethiopia's central highlands, is pushing the plant into increasingly inaccessible areas such as mountain edges, cliffs, and valleys.

### 4.5 The Potential Suitable Habitat for the Conservation of *B. macroptera*

The synergy between anthropogenic activities and climate change influences species distribution and abundance, particularly affecting the survival of endemic species (Jun and Yongyong 2008; Seifollahi‐Aghmiuni et al. 2022). Consequently, a significant portion of *B. macroptera*'s natural habitat is vulnerable to further loss and fragmentation. Although *B. macroptera* naturally grows abundantly and disperses its seeds effectively (Girmay et al. 2022), effective conservation measures are still necessary.

Effective landscape protection and restoration rely on integrating species distribution modeling findings (Sinclair et al. 2010). This approach identifies suitable habitats and essential requirements, enabling targeted conservation efforts. *Bidens macroptera* is expected to remain suitable and expand into new areas of subafroalpine and afroalpine zones. These regions are high priority for conservation due to their elevation and mountainous terrain, which are ideal for highland plants. The IUCN has recognized *B. macroptera* as an endemic species to Ethiopia and Eritrea, aiming to provide information on the status, trends, and threats to species, thereby guiding biodiversity conservation efforts (Noroozi et al. 2018). However, endemism does not necessarily correlate with species richness (Orme et al. 2005) and is insufficient for accurately assessing current and future distribution ranges and habitat suitability. Therefore, the habitat suitability results from this study are crucial for validating the IUCN Red List program over time and across different regions. Additionally, we have mapped the current suitable areas for conservation at a zonal level (Appendix C). This information is crucial for conserving the study species, particularly regarding ex situ and in situ habitat management, especially in high‐suitability areas.

## 5 Conclusion

Our prediction model indicates that *B. macroptera* is predominantly distributed across various provinces in Ethiopia. However, its suitable habitat is shrinking due to the combined impacts of climate change and human activities. The model forecasts that the central highlands, particularly the mountainous regions and near subafroalpine regions, will remain or emerge as suitable habitats under future climate scenarios. These habitats can be prioritized for conservation efforts to ensure the species' survival and promote biodiversity.

This study highlights the substantial influence of fluctuation in temperature and soil pH condition on the distribution and habitat suitability of *B. macroptera*. This knowledge serves as a starting guide to habitat restoration and management practices. We suggest an integrated conservation strategy, incorporating both in situ (e.g., in parts of Shewa, Gondar, and Gojam) and ex situ (e.g., cultivation in home gardens) approaches to rescue the species from further endangerment. Together, these strategies may enhance the species' resilience to ongoing climatic and anthropogenic pressures.

## Author Contributions

**Tsige Hailegiorgis:** conceptualization (lead), data curation (lead), formal analysis (lead), investigation (lead), methodology (equal), software (equal), validation (equal), visualization (lead), writing – original draft (lead), writing – review and editing (lead). **Debissa Lemessa:** software (equal), supervision (lead), validation (lead), writing – review and editing (equal). **Daniel Melese:** data curation (equal), software (equal). **Mikiyas Abebe:** data curation (equal), software (equal). **Sileshi Nemomissa:** software (equal), supervision (lead), validation (lead), writing – review and editing (equal).

## Conflicts of Interest

The authors declare no conflicts of interest.

## Data Availability

Data is available on Zenodo at https://zenodo.org/records/15849512 (DOI: https://doi.org/10.5281/zenodo.15849512).

## Appendix A Habitat Suitability Changes

**Table:**

Habitat suitability	Change type	Category	Description
Unsutable	Unsuitable—unsuitable	Unsuitable	Remains unsuitable across comparison scenarios
	Unsuitable—suitable	Gain in suitability	Areas that shift from unsuitable to suitable
	Suitable—unsuitable	Loss in suitability	Areas that shift from suitable to unsuitable
Suitable	Suitable—suitable	Stable	Remain suitable across all comparison scenarios
